# Toothbrushing and School Refusal in Elementary School: A Longitudinal Study

**DOI:** 10.3390/ijerph17207505

**Published:** 2020-10-15

**Authors:** Yoshifumi Fukuya, Yusuke Matsuyama, Aya Isumi, Satomi Doi, Manami Ochi, Takeo Fujiwara

**Affiliations:** 1 Department of Global Health Promotion, Tokyo Medical and Dental University, Tokyo 113-8519, Japan; yfukuyabgs@gmail.com (Y.F.); matsuyama-thk@umin.org (Y.M.); isumi.hlth@tmd.ac.jp (A.I.); doi.hlth@tmd.ac.jp (S.D.); 2 Japan Society for the Promotion of Science, Tokyo 102-0083, Japan; 3 National Institute of Public Health, Department of Health and Welfare Services, Saitama 351-0104, Japan; ochi.m.aa@niph.go.jp

**Keywords:** oral hygiene, mental health, epidemiology, biostatistics, prevention, behavioral science, child dentistry

## Abstract

The aim of this study was to examine the association between toothbrushing frequency and school refusal among elementary school children. We used data from the Adachi Child Health Impact of Living Difficulty (A-CHILD) longitudinal study conducted between 2015 and 2016 in Adachi City, Tokyo, Japan. A questionnaire was distributed to all first-grade children aged 6 to 7 years (N = 3697, follow-up rate: 86.2%). Propensity score (PS) matching was applied to collapse the known covariates on toothbrushing frequency in grade 1 on the association with school refusal in grade 2. Among the followed children, 2.4% showed school refusal in grade 2 (89 children) and 23.5% (870 children) brushed their teeth once or less than once daily in grade 1. After propensity score matching, children with toothbrushing once or less than once daily in grade 1 were 2.25 (95% CI: 1.25–4.05) times more likely to show school refusal in grade 2, compared with those with toothbrushing twice or more a day. Our findings suggest that toothbrushing once or less than once daily is an independent risk factor for school refusal among children. Oral health promotion to recommend toothbrushing more than once a day could prevent school refusal. Further intervention studies investigating the mechanism and causality are warranted.

## 1. Introduction

School refusal is a major school-related problem for children and adolescents [1]. School refusal refers to refusal or reluctance to attend school or problems staying in school, often causing prolonged absence. School refusal can be characterized as a manifestation of emotional problems, mainly depression and anxiety, and oppositional defiant disorder or conduct disorder [2]. Prior research has demonstrated the negative outcomes of school refusal, including impaired emotional and social development and poor academic achievement in childhood [3], and unemployment, marriage problems, and mental illness in adulthood [4]. Hence, it is worthwhile to elucidate risk factors for school refusal for the minimization and prevention of the short- and long-term consequences.

Oral health plays a central role in general health status and quality of daily life. Oral health problems in children may cause dental pain, discomfort, and impaired daily activities [5]. Furthermore, previous studies have indicated an association of poor oral health among elementary school children with lower levels of academic performance [6,7] and school attendance problems [5,8,9,10]. While these studies suggest that improving a child’s oral health may contribute to a reduction in adverse educational consequences, the causality remains unknown.

Toothbrushing is one of the most important methods to prevent dental problems, such as periodontal disease and dental caries, with the latter requiring adequate utilization of fluoride toothpaste [11,12,13]. Twice daily toothbrushing is widely recommended to maintain good oral health [14]. Although oral health behaviors in childhood are affected by a variety of factors such as parenting [15] and socioeconomic status [16], toothbrushing habits can be changed over time and are thus modifiable [17]. Indeed, several studies have reported that home- and school-based interventions to promote toothbrushing have positive effects on preventing dental caries [18] and on the oral health behavior of children [19]. Given the possible relationship between a child’s oral health and school-related problems, toothbrushing may be one of the intervention approaches for preventing school refusal. Notably, prior studies have indicated that toothbrushing frequency is associated with school performance in adolescents [20] and education level in adulthood [21]. To date, however, the relationship between toothbrushing frequency and school refusal remains unknown.

Therefore, the aim of our study was to examine the association between toothbrushing frequency and school refusal at age 7 to 8 years using a longitudinal population sample of school children. We used a propensity score (PS) approach for this analysis to minimize potential bias, controlling for several demographic, socioeconomic, parenting, child’s lifestyle, and mental health variables.

## 2. Materials and Methods

### 2.1. Participants

**Figure 1:**
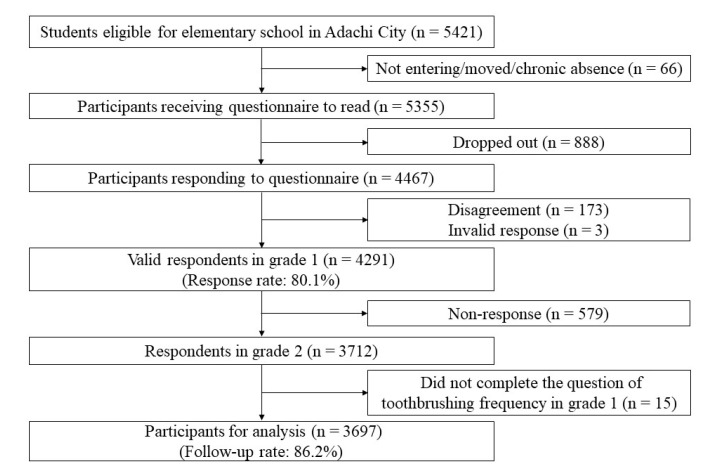
Flow chart of participants.

Data were obtained from the Adachi Child Health Impact of Living Difficulty (A-CHILD) longitudinal study conducted in all 69 public elementary schools in Adachi City, Tokyo, Japan [22]. In 2015, a questionnaire was distributed to all children aged 6 to 7 years in grade 1 (*n* = 5355). The questionnaire answered by caregivers was submitted to the school using an anonymous envelope (*n* = 4467). A total of 4291 caregivers provided informed consent (response rate: 80.1%). In 2016, we followed up with all the children aged 7 to 8 years in grade 2 (*n* = 3712). We excluded participants who did not answer the question about toothbrushing frequency in grade 1 (*n* = 15). After the exclusion, the number of participants who did not respond to the question about school refusal in grade 2 was zero. Finally, 3697 children were involved in both 2015 and 2016 surveys for analysis (follow-up rate: 86.2%) (Figure 1).

### 2.2. Measurements

#### 2.2.1. School Refusal

The caregivers were asked whether their child was absent from school in the six months since the beginning of the fiscal year, in grades 1 and 2. They were also asked reasons for the school absence using the following categories: (1) Illness or injury; (2) Family reasons; (3) He/She did not want to go to school; and (4) Other reasons. We defined the response of (3) as school refusal, and the cases of school refusal were dichotomized (0 = No or 1 = Yes).

#### 2.2.2. Toothbrushing Frequency

Caregivers were asked how many times their child performed toothbrushing in a day in grade 1 using the following items: “more than once a day”, “once a day”, and “not every day”. The items of “once a day” and “not every day” were classified into one category; then, toothbrushing frequency was assessed as a binary variable (0 ≥ twice a day or 1 ≤ once a day).

#### 2.2.3. Parental Involvement with Child

This score was assessed based on the frequency of nine types of parental involvement with their child (tutoring; playing activities; playing computer games; playing cards games; talking about school life; talking about news; talking about TV programs; preparing meals; and going out) in grade 1. We summed the frequency of each item using the following item responses: 0 “seldom”, 1 “once or twice per month”, 2 “once or twice per week”, 3 “three or four times per week”, and 4 “almost every day” (Cronbach’s alpha = 0.72), and categorized them into tertile (1 = low, 2 = middle, or 3 = high).

#### 2.2.4. Maltreatment

Child maltreatment, that is, physical abuse, neglect, and psychological abuse, was assessed by eight items that were adopted from 17 items of the Japanese child maltreatment scale (α = 0.77) [23]. Physical abuse, neglect, and psychological abuse were measured using two, three, and three items out of them, respectively. The 4-point Likert scale for each item was used as follows: 1 = “often”, 2 = “sometimes”, 3 = “rarely”, and 4 = “not at all”. These responses were dichotomized (“yes” or “no”) based on expert review from the viewpoint of the severity and frequency of maltreatment in Japan [24]. When any item in each category of maltreatment had a “yes” response at least once, we dichotomized the category into 1 = “Yes” and 0 = “No”. The details of the procedure were described elsewhere [24].

#### 2.2.5. Lifestyle

Caregivers were asked about their child’s lifestyle in glade 1, using nine items including eating habits (the frequencies of drinking juice, having breakfast, and eating vegetables and snacks), frequency of physical activities, time spent on watching TV and playing a game, and bedtime and wake-up time. The responses in each item were dichotomized (Table 1).

#### 2.2.6. Child Mental Health

Child emotional and behavior problems, that is, emotional symptoms, conduct problems, hyperactivity/inattention, and peer problems, in grade 1 was assessed using the scales of total difficulties score from the Japanese version of the Strengths and Difficulties Questionnaire (SDQ) [25]. The reliability and validity of the SDQ in Japanese children were reported in prior research [26].

Child resilience in grade 1 was assessed based on the Children’s Resilient Coping Scale (CRCS) [27]. The scale consisted of eight items with high internal consistency (Cronbach’s alpha = 0.80). The caregivers rated child resilience and coping behavior using a scale of 0 “never” to 4 “very frequently”. The total score of the CRCS was calculated from the summed score of the eight items (range: 0–32); higher total scores indicated higher resilience. A score <10th percentile was defined as low resilience and dichotomized (0 = Not Low or 1 = Low).

### 2.3. Other Variables

Caregivers were asked about child sex, birth order (only child, eldest, youngest, or middle), marital status, and household income. The caregiver’s psychological distress was measured by using the Japanese version of the Kessler 6 (K6) when their child was in grade 1 [28]. It consists of six items related to depression and anxiety, rated on a 5-point Likert scale. The total score of the items was calculated (range: 0–24); a score of 5–12 was defined as moderate psychological distress and a score of ≥13 was defined as severe [29].

### 2.4. Statistical Analysis

First, we conducted multivariate logistic regression analysis to examine the association of toothbrushing frequency in grade 1 (reference: ≥twice a day) with school refusal in grade 2 as the following models: Model 1 was adjusted for child sex, parental marital status, siblings, household income, and caregiver’s K6 score; Model 2 added all variables of parenting (parental involvement with child, neglect, physical abuse, and psychological abuse) and lifestyle (*p* < 0.05) to Model 1; Model 3 was additionally adjusted for school refusal in grade 1; and Model 4 was further adjusted for the SDQ and the CRCS in grade 1. Missing data were substituted by dummy variables.

Second, propensity score (PS) matching was conducted to compare the differences in the characteristics between children with toothbrushing frequency twice or more a day and those with once or less than once a day. Individual propensity scores were calculated by logistic regression modeling that incorporated the following 21 variables measured in grade 1: child sex, marital status, siblings, household income, caregiver’s K6, parenting, lifestyle, mental health, and school refusal. We conducted a 1:1 optimal propensity score matching within a 0.01 caliper width without replacement. The balance of covariates between the matched pairs was tested based on standardized biases, which was less than 10% in all variables and not significant in a chi-squared test (Table 1). Of the children with toothbrushing once or less than once a day, 835 children (96.0%) were matched to similar children with toothbrushing twice or more a day. Using the matched pairs, conditional logistic regression analysis was conducted to examine the association of toothbrushing frequency in grade 1 with school refusal in grade 2. We used STATA version 15.0 (StataCorp, College Station, TX, USA) for all analysis and followed the Strengthening the Reporting of Observational Studies in Epidemiology Statement (STROBE) guidelines.

### 2.5. Ethics Statement

This study was approved by the Ethics Committee at the National Center for Child Health and Development (Study ID: 1147) and Tokyo Medical and Dental University (Study ID: M2016-284), Tokyo, Japan.

## 3. Results

Table 1 shows the characteristics of children in grade 1. Among the eligible 3697 children, 870 children (23.5%) reported their toothbrushing frequency to be once or less than once a day. School refusal in grade 2 was reported by 89 (2.4%) children. Before PS matching, there was a significant difference in marital status, household income, caregiver’s K6 score, and school refusal in grade 1. As for parenting, the percentage of parental involvement with child was lower in children with toothbrushing once or less than once a day. The percentage of neglect, physical abuse, and psychological abuse was higher in children with toothbrushing once or less than once a day (*p <* 0.05). Children with toothbrushing once or less than once a day showed a less healthy lifestyle including higher frequency of drinking juice and eating snacks, lower frequency of having breakfast and doing exercise, and more hours of watching TV and playing a game (*p <* 0.05). Bedtime and wake-up time were later in children with toothbrushing once or less than once a day (*p <* 0.05). As for mental health, these children showed more difficulties in the total score of the SDQ (*p <* 0.05) and lower resilience in the total score of the CRCS (*p <* 0.05). After PS matching, there was no difference in all variables between both groups.

Table 2 shows the results of multivariate logistic regression analysis before PS matching. Model 1, adjusted for child sex, parental marital status, siblings, household income, and caregiver’s K6, showed that toothbrushing once or less than once a day in grade 1 had an increased risk of school refusal in grade 2 (odds ratio (OR) = 2.61, 95% confidence interval (CI): 1.70–3.99). In Model 2 (all items of parenting and lifestyles in grade 1 added to Model 1), children with toothbrushing once or a less than once a day in grade 1 were 2.32 times (95% CI: 1.50–3.58) more likely to show school refusal in grade 2 than those with toothbrushing twice or more a day. In Model 3, even after being adjusted for school refusal in grade 1, toothbrushing once or a less than once a day in grade 1 had an increased risk of school refusal in grade 2 (OR = 1.76, 95% CI: 1.76–2.90). In Model 4, adjusted for the total scores of the SDQ and the CRCS in grade 1, a significant association of toothbrushing once or less than once a day in grade 1 with school refusal in grade 2 remained (OR = 1.66, 95% CI: 1.00–2.76).

Table 3 shows the association of toothbrushing frequency in grade 1 with school refusal in grade 2 after PS matching. The number of school refusals in grade 2 was 19 (2.3%) in children with toothbrushing twice or more a day and 36 (4.3%) in those with once or less than once a day. Odds ratio of school refusal in grade 2 for children with once or less than once a day in grade 1 was 2.25 (95% CI = 1.25–4.05) when those with twice or more a day was considered as a reference.

## 4. Discussion

Our study examined whether toothbrushing frequency was associated with school refusal among elementary school children. We found that children with low frequency of toothbrushing in grade 1 showed an increased risk of school refusal in grade 2. To the best of our knowledge, this is the first longitudinal study to examine the association between the frequency of toothbrushing and school refusal among children.

There are several possible mechanisms that explain why toothbrushing frequency is prospectively associated with school refusal among children. First, infrequent toothbrushing can induce impairment of oral functions due to oral health problems such as caries and periodontal diseases [11,13]. Functional problems such as pain, discomfort, and difficulties of mastication reduce one’s quality of life [30]. These problems can also induce physical and social impairment of oral functions, such as difficulties in breathing, feeding, and speaking [5], which in turn cause physical and psychological stress to children [31], and may lead to school refusal. Indeed, prior research reported that poor oral conditions were associated with lower levels of academic performance together with reduced psychosocial well-being [7] and school attendance problems [5,8,9,10].

Second, infrequent toothbrushing can induce psychosocial stress through unpleasant oral conditions [32]. Previous research has reported that halitosis was associated with an increased risk of depression [33]. Furthermore, the undesired change of dental appearance due to infrequent toothbrushing, such as stain, can be a factor for being teased and bullied by peers [34,35], which may induce school refusal for children.

Third, poor oral hygiene due to infrequent toothbrushing can cause periodontal diseases such as gingivitis and periodontitis [11,13]. Invasion of periodontal pathogens, endotoxins, such as lipopolysaccharide (LPS), and pro-inflammatory cytokines, such as interleukin (IL)-1, IL-6, and tumor necrosis factor alpha (TNF), from local inflammation into blood circulation can induce systematic inflammation [36,37]. In rodent research, the injection of LPS and pro-inflammatory cytokines was found to provoke depression-like behavior [38,39]. Furthermore, systematic inflammation can lead to an increased risk of neuroinflammation, oxidative, and nitrosative stress, which may increase vulnerability to depression [40]. Notably, depression is one of the frequent comorbid conditions for school refusal [2]. Moreover, among periodontal diseases, gingivitis is a common oral health problem among children and adolescents worldwide [41]. Indeed, gingivitis is highly prevalent in Japanese children; 35.5% of children aged 5 to 9 years and 45.3% of those aged 10 to 14 years have symptoms of gingivitis [42]. Thus, infrequent toothbrushing may increase the risk of depression by exacerbating periodontal inflammation not only in adults but also in children, which in turn may induce school refusal. However, there is no empirical study that ascertains the association between these mechanisms and school refusal. Therefore, further longitudinal studies are needed to examine whether oral health problems caused by infrequent toothbrushing are associated with school refusal.

There are several limitations in this study. First, caregivers assessed both children’s toothbrushing and school refusal, which may result in common method bias. Thus, further study that objectively assesses toothbrushing frequency and school refusal by linking school records is needed. Second, we did not assess the reasons for school refusal. Previous research reported that children have several reasons for school refusal, such as academic achievement, peer-relationship problems, and school environment [4]. The association between toothbrushing frequency and school refusal may be different for each of the reasons. Hence, further assessment is needed to examine the association between the reasons and toothbrushing frequency, which may allow the understanding of the mechanisms of school refusal. Third, this study did not assess a child’s predisposition. A prior study found that elementary school children with lower self-control were more likely to have lower frequency of toothbrushing [43]. In addition, previous research has demonstrated that self-esteem was associated with toothbrushing frequency in young adolescents [44]. As a child’s predisposition may affect toothbrushing behavior, further longitudinal studies are needed to reveal whether specific predispositions of children are associated with school refusal. We applied PS matching to reduce the bias due to known variables on the allocation of toothbrushing. However, we did not measure other factors related to parents, such as parenting styles, and child lifestyles affecting the association between child toothbrushing frequency and school refusal. Nevertheless, the results may be biased by such unmeasured confounders, which were not able to be addressed in the analysis. Further randomized controlled trials to test the efficacy of toothbrushing on the prevention of school refusal is needed.

Despite these limitations, our current study demonstrated an independent association between toothbrushing frequency and school refusal with a longitudinal data and PS matching method among elementary school children in Japan. This finding suggests that oral health promotion recommending toothbrushing twice a day could prevent school refusal. To increase the frequency of toothbrushing, school teachers may need to consider implementing toothbrushing after school lunch as a school oral health promotion policy.

## 5. Conclusions

In conclusion, we found that infrequent toothbrushing in grade 1 was associated with school refusal in grade 2. Home- and school-based interventions for regular toothbrushing may reduce school refusal. Further randomized controlled trials to test the efficacy of toothbrushing on the prevention of school refusal are needed to prove the causality and mechanism of the current findings.

## Figures and Tables

**Table 1:** Demographic characteristics for toothbrushing frequency in grade 1 before and after propensity score (PS) matching.

Variables		Before PS Matching (N, %)				After PS Matching (N, %)			
		Toothbrushing ≥twice a day (N = 2827)	Toothbrushing ≤once a day (N = 870)	*p* *	Bias (%)	Toothbrushing ≥twice a day (N = 835)	Toothbrushing ≤once a day (N = 835)	*p* *	Bias (%)
Sex	Boys	1422 (50.3)	471 (54.1)	0.13		439 (52.6)	453 (54.3)	0.68	
	Girls	1403 (49.6)	398 (45.8)		−7.7	394 (47.2)	381 (45.6)		−3.1
	Missing	2 (0.1)	1 (0.1)		1.4	2 (0.2)	1 (0.1)		−3.9
Marital status	Married	2563 (90.7)	761 (87.5)	0.02		739 (88.5)	735 (882)	0.94	
	Single/Others	201 (7.1)	86 (9.9)		9.9	74 (8.9)	78 (9.3)		1.7
	Missing	63 (2.2)	23 (2.6)		2.9	22 (2.6)	22 (2.6)		0.0
Siblings	No siblings	620 (21.9)	147 (16.9)	<0.001		137 (16.4)	142 (17)	0.96	
	Eldest	932 (33.)	259 (29.8)		−7.0	247 (29.6)	252 (30.2)		1.3
	Youngest	992 (35.1)	348 (40.0)		10.1	343 (41.1)	333 (39.9)		−2.5
	Middle	283 (10.0)	116 (13.3)		10.3	108 (12.9)	108 (12.9)		0.0
	Missing	0 (0.0)	0 (0.0)		-	0 (0.0)	0 (0.0)		-
Household income	<3.0	289 (10.2)	107 (12.3)	0.002		97 (11.6)	101 (12.1)	0.98	
(million yen)	3.0 to <6.0	1125 (39.8)	362 (41.6)		3.6	343 (41.1)	345 (41.3)		0.5
	6.0 to <10.0	910 (32.2)	232 (26.7)		−12.1	230 (27.5)	226 (27.1)		−1.1
	≥10.0	252 (8.9)	67 (7.7)		−4.3	71 (8.5)	65 (7.8)		−2.6
	Missing	251 (8.9)	102 (11.7)		9.3	94 (11.3)	98 (11.7)		1.6
K6	<5	2057 (72.8)	561 (64.5)	<0.001		563 (67.4)	548 (65.6)	0.87	
	5 to <13	640 (22.6)	243 (27.9)		12.2	219 (26.2)	229 (27.4)		2.8
	≥13	105 (3.7)	54 (6.2)		11.5	42 (5)	47 (5.6)		2.8
	Missing	25 (0.9)	12 (1.4)		4.7	11 (1.3)	11 (1.3)		0.0
Parenting									
Parental involvement	Low	981 (34.7)	351 (40.3)	0.001		324 (38.8)	333 (39.9)	0.88	
	Middle	1000 (35.4)	319 (36.7)		2.7	324 (38.8)	308 (36.9)		−4.0
	High	832 (29.4)	196 (22.5)		−15.8	183 (21.9)	190 (22.8)		1.9
	Missing	14 (0.5)	4 (0.5)		−0.5	4 (0.5)	4 (0.5)		0.0
Neglect	No	2450 (86.7)	714 (82.1)	0.003		692 (82.9)	695 (83.2)	0.83	
	Yes	354 (12.5)	148 (17.0)		12.6	137 (16.4)	132 (15.8)		−1.7
	Missing	23 (0.8)	8 (0.9)		1.1	6 (0.7)	8 (1)		2.6
Physical abuse	No	2495 (88.3)	717 (82.4)	<0.001		719 (86.1)	699 (83.7)	0.36	
	Yes	305 (10.8)	147 (16.9)		17.8	112 (13.4)	130 (15.6)		6.3
	Missing	27 (1.0)	6 (0.7)		−2.9	4 (0.5)	6 (0.7)		2.7
Psychological abuse	No	1970 (69.7)	563 (64.7)	0.014		544 (65.2)	545 (65.3)	0.97	
	Yes	828 (29.3)	298 (34.3)		10.7	283 (33.9)	281 (33.7)		−0.5
	Missing	29 (1.0)	9 (1.0)		0.1	8 (1)	9 (1.1)		1.2
Lifestyle									
Drinking juice	Not every day	2086 (73.8)	595 (68.4)	0.001		566 (67.8)	578 (69.2)	0.60	
	Every day	492 (17.4)	202 (23.2)		14.4	184 (22)	184 (22)		0.0
	Missing	249 (8.8)	73 (8.4)		−1.5	85 (10.2)	73 (8.7)		−5.1
Having breakfast	Every day	2743 (97.0)	771 (88.6)	<0.001		771 (92.3)	762 (91.3)	0.42	
	Not every day	81 (2.9)	99 (11.4)		33.5	64 (7.7)	73 (8.7)		4.2
	Missing	3 (0.1)	0 (0)		-	0 (0.0)	0 (0.0)		-
Eating vegetables	Every day	2334 (82.6)	675 (77.6)	<0.001		641 (76.8)	656 (78.6)	0.54	
	Not every day	243 (8.6)	120 (13.8)		16.5	107 (12.8)	105 (12.6)		−0.8
	Missing	250 (8.8)	75 (8.6)		−0.8	87 (10.4)	74 (8.9)		−5.5
Eating snacks	At a fixed time	1986 (70.3)	476 (54.7)	<0.001		446 (53.4)	472 (56.5)	0.37	
	Anytime	588 (20.8)	321 (36.9)		36.1	304 (36.4)	290 (34.7)		−3.8
	Missing	253 (9.0)	73 (8.4)		−2.0	85 (10.2)	73 (8.7)		−5.1
Doing exercise per week	≥one time	2586 (91.5)	752 (86.4)	<0.001		726 (87)	732 (87.7)	0.74	
	Rarely or none	238 (8.4)	116 (13.3)		15.8	108 (12.9)	101 (12.1)		−2.7
	Missing	3 (0.1)	2 (0.2)		3.0	1 (0.1)	2 (0.2)		2.9
Watching TV per day	<3 h	2514 (88.9)	727 (83.6)	<0.001		715 (85.6)	709 (84.9)	0.92	
	≥3 h	308 (10.9)	140 (16.1)		15.2	117 (14)	123 (14.7)		2.1
	Missing	5 (0.2)	3 (0.3)		3.3	3 (0.4)	3 (0.4)		0.0
Playing a game per day	<1 h	2282 (80.7)	629 (72.3)	<0.001		615 (73.7)	608 (72.8)	0.89	
	≥1 h	530 (18.8)	236 (27.1)		20.1	216 (25.9)	222 (26.6)		0.6
	Missing	15 (0.5)	5 (0.6)		1.7	4 (0.5)	5 (0.6)		1.6
Bedtime	≤10 PM	2368 (83.8)	606 (69.7)	<0.001		597 (71.5)	604 (72.3)	0.92	
	>10 PM	304 (10.8)	144 (16.6)		16.9	140 (16.8)	134 (16.1)		−2.1
	Missing	155 (5.5)	120 (13.8)		28.4	98 (11.7)	97 (11.6)		−0.4
Wake-up time	≤7 AM	1607 (56.8)	362 (41.6)	<0.001		343 (41.1)	358 (42.9)	0.51	
	>7 AM	1176 (41.6)	481 (55.3)		27.6	473 (56.7)	453 (54.3)		−4.8
	Missing	44 (1.6)	27 (3.1)		10.3	19 (2.3)	24 (2.9)		4.0
Mental health									
SDQ: Total Difficulties Score	Normal	2091 (74.0)	547 (62.9)	<0.001		549 (65.8)	539 (64.6)	0.89	
	Borderline	362 (12.8)	127 (14.6)		5.3	126 (15.1)	123 (14.7)		−1.0
	Clinical	351 (12.4)	187 (21.5)		24.3	152 (18.2)	164 (19.6)		3.9
	Missing	23 (0.8)	9 (1.0)		2.3	8 (1)	9 (1.1)		1.3
CRCS: Total Score	Not Low	2619 (92.6)	736 (84.6)	<0.001		725 (86.8)	720 (86.2)	0.89	
	Low	203 (7.2)	129 (14.8)		24.6	107 (12.8)	111 (13.3)		1.5
	Missing	5 (0.2)	5 (0.6)		6.5	3 (0.4)	4 (0.5)		2.0
School refusal									
School refusal in grade 1	No	2763 (97.7)	838 (96.3)	0.04		812 (97.3)	808 (96.8)	0.84	
	Yes	56 (2.0)	30 (3.5)		9.0	21 (2.5)	25 (3)		2.9
	Missing	8 (0.3)	2 (0.2)		−1.1	2 (0.2)	2 (0.2)		0.0

* *p*-value for chi-squared test. SDQ, Strengths and Difficulties Questionnaire; CRCS, Children’s Resilient Coping Scale.

**Table 2:** Association of toothbrushing frequency with school refusal in logistic regression analysis.

Variables		Crude OR (95% CI)	Model 1 ^a^ OR (95% CI)	Model 2 ^b^ OR (95% CI)	Model 3 ^c^ OR (95% CI)	Model 4 ^d^ OR (95% CI)
Toothbrushing frequency	≥twice a day	Ref	Ref	Ref	Ref	Ref
	≤once a day	2.61 *** (1.70–3.99)	2.32 *** (1.50–3.58)	1.89 ** (1.19–3.00)	1.76 * (1.06–2.90)	1.66 * (1.00–2.76)
Parenting						
Parental involvement	Low	Ref	Ref	Ref	Ref	Ref
	Middle	0.80 (0.49–1.29)	0.85 (0.52–1.37)	0.89 (0.54–1.46)	0.84 (0.50–1.43)	0.92 (0.54–1.57)
	High	0.62 (0.36–1.09)	0.72 (0.41–1.27)	0.83 (0.46–1.49)	0.83 (0.45–1.53)	0.95 (0.51–1.77)
Neglect	No	Ref	Ref	Ref	Ref	Ref
	Yes	2.25 ** (1.39–3.66)	1.76 * (1.06–2.91)	1.46 (0.86–2.49)	1.09 (0.60–1.96)	1.07 (0.59–1.93)
Physical abuse	No	Ref	Ref	Ref	Ref	Ref
	Yes	2.25 ** (1.37–3.71)	1.58 (0.93–2.66)	1.18 (0.66–2.12)	1.24 (0.66–2.32)	1.19 (0.63–2.23)
Psychological abuse	No	Ref	Ref	Ref	Ref	Ref
	Yes	1.96 ** (1.28–2.99)	1.45 (0.93–2.26)	1.18 (0.72–1.94)	1.22 (0.72–2.07)	1.15 (0.68–1.97)
Lifestyle						
Drinking juice	Not every day	Ref	Ref	Ref	Ref	Ref
	Every day	1.97 ** (1.24–3.13)	1.81 * (1.13–2.89)	1.56 (0.96–2.54)	1.52 (0.90–2.57)	1.49 (0.88–2.51)
Having breakfast	Every day	Ref	Ref	Ref	Ref	Ref
	Not every day	3.52 *** (1.92–6.47)	2.79 ** (1.47–5.27)	1.64 (0.83–3.24)	1.24 (0.57–2.69)	1.20 (0.55–2.60)
Eating vegetables	Every day	Ref	Ref	Ref	Ref	Ref
	Not every day	2.33 ** (1.37–3.96)	1.94 * (1.12–3.36)	1.40 (0.79–2.48)	1.37 (0.73–2.56)	1.24 (0.66–2.36)
Eating snacks	At a fixed time	Ref	Ref	Ref	Ref	Ref
	Anytime	1.82 ** (1.16–2.84)	1.74 * (1.10–2.75)	1.19 (0.73–1.94)	1.08 (0.63–1.83)	1.01 (0.59–1.73)
Doing exercise per week	≥one time	Ref	Ref			
	Rarely or none	1.20 (0.62–2.34)	1.06 (0.54–2.08)			
Watching TV per day	<3 h	Ref	Ref	Ref	Ref	Ref
	≥3 h	3.11 *** (1.95–4.96)	2.67 *** (1.65–4.30)	2.11 ** (1.28–3.49)	1.74 * (1.00–3.00)	1.70 (0.98–2.97)
Playing a game per day	<1 h	Ref	Ref			
	≥1 h	1.68 * (1.06–2.66)	1.39 (0.86–2.26)			
Bedtime	≤10 PM	Ref	Ref			
	>10 PM	1.59 (0.86–2.93)	1.54 (0.83–2.86)			
Wake-up time	≤7 AM	Ref	Ref			
	>7 AM	1.35 (0.87–2.07)	1.30 (0.84–2.01)			
School refusal						
School refusal in grade 1	No				Ref	Ref
	Yes				17.6 *** (9.68–32.1)	16.4 *** (8.96–30.0)
Mental health						
SDQ: Total Difficulties Score	Normal					Ref
	Borderline					1.11 (0.58–2.16)
	Clinical					1.22 (0.66–2.26)
CRCS: Total Score	Not Low					Ref
	Low					1.82 (0.99–3.36)

OR: odds ratio; 95% CI: 95% confidence interval. *** *p* < 0.001, ** *p* < 0.01, * *p* < 0.05. ^a^ Model 1: adjusting for child sex, parental marital status, siblings, household income, and K6. ^b^ Model 2: adding parenting and lifestyle (*p* < 0.05) in grade 1 to Model 1. ^c^ Model 3: adding school refusal in grade 1 to Model 2. ^d^ Model 4: adding the Children’s Resilient Coping Scale (CRCS) and the Strength and Difficulties Questionnaire (SDQ) to Model 3.

**Table 3:** Association of toothbrushing frequency with school refusal after propensity score matching.

Number of School Refusals in Grade 2 (N, %)		OR (95% CI)	*p*-Value
Toothbrushing Frequency in Grade 1 ≥ Twice a Day	Toothbrushing Frequency in Grade 1 ≤ Once a Day		
16 (1.9)	36 (4.3)	2.25 (1.25–4.05)	0.007

OR: odds ratio; 95% CI: 95% confidence interval.
